# Increased nuchal translucency and pregnancy outcomes: experience of Başkent University Ankara Hospital

**DOI:** 10.4274/tjod.galenos.2019.51482

**Published:** 2019-07-03

**Authors:** Nihal Şahin Uysal, Çağrı Gülümser, Zerrin Yılmaz Çelik, Filiz Bilgin Yanık

**Affiliations:** 1 Başkent University Faculty of Medicine, Department of Obstetrics and Gynecology, Ankara, Turkey; 2 Başkent University Faculty of Medicine, Department of Medical Genetics, Ankara, Turkey

**Keywords:** Nuchal translucency, abnormal karyotype, cardiac anomaly

## Abstract

**Objective:**

First trimester nuchal translucency (NT) measurement is considered to be an important tool in antenatal follow-up. This study aimed to evaluate the outcomes of pregnancies with increased NT at Başkent University Ankara Hospital between 2004 and 2016.

**Materials and Methods:**

Patients with NT measurements ≥1.5 multiples of median (MoM) were divided into two groups; group I included increased NT cases without fetal anomalies (either abnormal fetal karyotype or congenital structural anomalies) or loss (intrauterine fetal death), and group II included increased NT cases with fetal anomalies or loss. The groups were compared with each other with respect to maternal demographic features and NT measurements.

**Results:**

Karyotype analyses were normal in 73.1% of cases with increased NT (57/78). Among those, 21.1% (12/57) had structural anomalies, and to specify, 9.6% (5/52 over 18 weeks) had cardiac anomalies. Although maternal demographic features did not differ significantly, NT measurements, both as millimeters and MoM, were significantly higher in group II (p<0.05). According to the receiver operating characteristic (ROC) curves, the optimal cut-off values for NT measurements for predicting fetal anomalies or loss were 3.05 mm and 2.02 MoM. NT measurement >7 millimeters or NT MoM >4.27 resulted in poor fetal outcomes without exception.

**Conclusion:**

Higher NT measurements indicate poorer pregnancy outcomes. Our study indicates that fetal echocardiography must be considered for all cases with increased NT.

**PRECIS:** First trimester nuchal translucency measurement is an important tool to recognize genetic abnormalities and many structural anomalies.

## Introduction

Nuchal translucency (NT) is the sonographic visualization of subcutaneous fluid accumulation behind the fetal neck in the first trimester. Ultrasonographic NT measurement at 11-13^6^ gestational weeks of pregnancy was introduced into clinical practice in the 1990s as a new method of aneuploidy screening, combined with maternal age and serum biochemical markers^(1)^. NT is supposed to be increased when it is ≥95^th^ percentile or ≥1.5 multiples of median (MoM) for a certain crown-rump length (CRL) measurement. Although the definitions may vary in the literature, any value ≥3.5 mm is ≥99^th^ percentile for any gestational age between 11-13^6^ weeks and considered to be absolutely abnormal, requiring fetal karyotyping as well as detailed anatomic examination with fetal echocardiography in the second trimester.

NT was primarily regarded as a marker of trisomies 21, 18, and 13, and Turner syndrome; however, increasing evidence suggests that abnormal NT measurements might also be related to other chromosomal abnormalities and genetic syndromes, structural anomalies, skeletal dysplasia, congenital infections, metabolic and hematologic disorders and other rare conditions^(2)^. The proposed mechanisms underlying increased NT, whether septated or not, are cardiac dysfunction, abnormal lymphatic drainage, venous congestion in the head and neck, altered composition of the extracellular matrix, fetal anemia, fetal hypoproteinemia, and fetal infection^(3)^. Increased NT may also be associated with generalized fetal hydrops^(4)^.

The aim of this study was to evaluate the outcomes of pregnancies with increased NT detected at 11-13^6^ gestational weeks at Başkent University Ankara Hospital from 2004 to 2016. We also aimed to consider these results in association with previous evidence to improve counseling for patients with increased NT.

## Materials and Methods

This study was approved by the Institutional Review Board and Ethics Committee of Başkent University (approval no: KA09/296). We reviewed the perinatology records of Başkent University Ankara Hospital from 2004 to 2016 to identify pregnancies with increased NT. Patients’ files were reviewed to determine maternal demographic features, results of karyotype analysis, incidence and types of fetal anomalies, and pregnancy outcomes. Cases without karyotype analyses were excluded.

NT measurement was performed as originally described by the Fetal Medicine Foundation^(1)^. The NT measurement in millimeters was converted to MoM according to the CRL, using the NT calculator program of the Fetal Medicine Foundation. Any value ≥1.5 MoM was defined as increased NT. Maternal serum antibody screening was performed for congenital infections including toxoplasma, cytomegalovirus, rubella, and parvovirus B19. Fetal anomaly scanning was performed after the 18^th^ gestational week.

Study cases were categorized in two groups after excluding cases with unknown pregnancy outcomes: group I included cases of increased NT without fetal anomalies (either abnormal fetal karyotype or congenital structural anomalies) or loss (intrauterine fetal death), and group II included increased cases of NT with fetal anomalies or loss. The two groups were compared with respect to maternal age, gravidity, parity, CRL at the time of NT measurement, and NT measurements.

### Statistical Analysis

Statistical analysis were performed using Student’s t-tests or the Mann-Whitney U test where appropriate, using the SPSS program. The results were considered to be statistically significant at p<0.05. The rates of preterm delivery, small for gestational age (SGA) and large for gestational age (LGA) newborns in group I were calculated. Preterm delivery was accepted as deliveries before 37 gestational weeks. SGA was defined as birth weight below the 10^th^ percentile, and LGA was defined as birth weight above the 90^th^ percentile.

The receiver operating characteristic (ROC) curve for NT measurements was drawn using SPSS to determine the optimal cut-off values for discriminating between groups I and II.

## Results

A total of 94 pregnant women underwent invasive diagnostic procedures because of increased NT from 2004 to 2016. Maternal serum screening for congenital infections was negative in all cases. Out of the 94 women, 78 with known pregnancy outcomes were included in the study. Among these 78 pregnancies, 74 were singletons (94.9%), 3 were twin pregnancies, and 1 was a triplet pregnancy. Selective fetocide was performed in 2 of the multiple pregnancies for a hydropic co-twin with trisomy 21 and a co-triplet with structural anomalies, respectively.

The mean maternal age of the study group (n=78) was 31.5±6.05 (range, 20-47) years. The mean gravidity was 1.82±1.21 (range, 1-6), the mean parity was 0.45±0.69 (range, 0-3), the mean NT measurements were 2.62±1.31 (range, 1.5-6.76) MoM, and 3.93±2.0 (range, 2-10) mm. Among the 78 pregnancies, 34 (43.6%) had NT measurements ≥3.5 mm.

**Figure 1:**
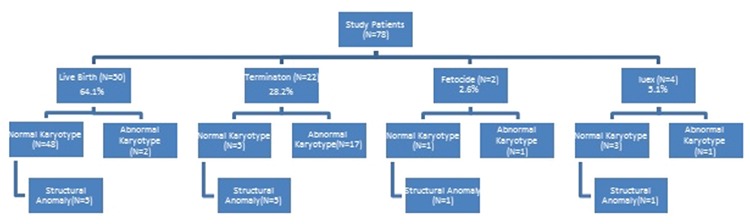
Fetal outcomes in cases undergoing invasive diagnostic procedures because of increased first-trimester nuchal translucency

Karyotype analyses were reported to be abnormal in 21 (26.9%) and normal in 57 (73.1%) of the 78 cases of increased NT. Among the entire study group, 50 (64.1%) pregnancies resulted in live births, 22 (28.2%) were terminated, selective fetocide was performed in 2 (2.6%), and intrauterine exitus occurred in 4 (5.1%). Abnormal karyotype results were present in 2 live births, 17 terminations, 1 selective fetocide, and 1 intrauterine exitus case. Structural defects with normal karyotype results were present in 5 live births, 5 terminations, 1 selective fetocide, and 1 intrauterine exitus case (Figure 1) (Table 1).

There were 43 cases of increased NT without fetal anomalies or loss (group I) and 35 increased NT cases with fetal anomalies or loss (group II). Group II comprised 21 (26.9%) cases with abnormal karyotype results: trisomy 21 in 9 (11.5%), trisomy 18 in 4 (5.1%), Turner syndrome in 6 (7.7%), deletion of 7q in 1 (1.3%), and duplication of 4p in 1 (1.3%). Twelve (15.3%) patients in group II had fetal structural anomalies despite normal karyotype: seven cases had multiple anomalies including 2 with cardiac anomalies, 3 had isolated cardiac anomalies, 1 had cleft lip and palate, and 1 had uretero-pelvic stenosis. Of the 52 pregnancies with normal karyotypes that continued beyond 18 weeks of gestation, cardiac anomalies were detected in 5 cases (9.6%), including aortic coarctation, complex cardiac anomalies, and ventricular septal defects in 1, 2, and 2 fetuses, respectively. One of these 5 fetuses had an NT measurement <3.5 mm (2.4 mm). Intrauterine exitus with no detected abnormality was observed in 2 pregnancies (Table 1).

**Figure 2:**
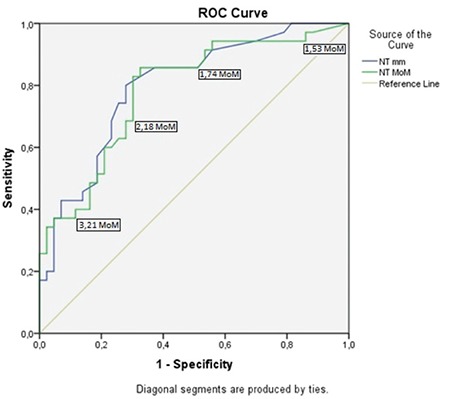
ROC curves of nuchal translucency measurements as MoM and millimeters for the whole study group (n=78) ROC: Receiver operating characteristic, MoM: Multiples of median, NT: Nuchal translucency

There was no significant difference in maternal age, gravidity, parity, or CRL at the time of NT measurement between groups I and II. However, NT measurements, both as millimeters and MoM, were significantly higher in group II (p<0.05) (Table 2). According to the ROC curves, the optimal cut-off values for NT measurements for predicting fetal anomalies or loss were 3.05 mm and 2.02 MoM (Figure 2). In our study population, all cases with NT measurements >7 mm or NT MoM >4.27 were associated with fetal anomalies or loss.

**Figure 3:**
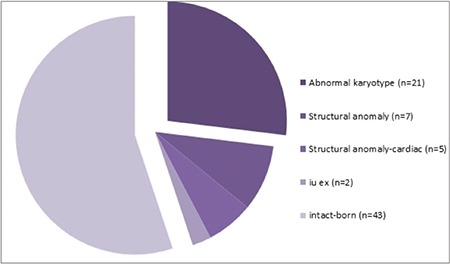
Increased nuchal translucency (NT) and perinatal outcomes [increased NT >1.5 MoM (n=78)] MoM: Multiples of median

The rates of preterm birth, SGA and LGA newborns in the increased NT group without fetal anomalies or loss (group I) were 13.9%, 13.9%, and 4.7%, respectively. Pregnancy outcomes in cases of increased NT and normal karyotype can be observed in Table 1. Perinatal outcomes of the all study participants are summarized in Figure 3.

## Discussion

The measurement of fetal NT between 11 and 13^6^ weeks of gestation is used as a screening method for chromosomal abnormalities in general practice. In fact, besides chromosomal abnormalities, increased NT may be due to several other problems^(2)^. Higher NT measurements indicate poorer outcomes and are usually associated with chromosomal defects and structural anomalies^(5,6,7)^.

Pan et al.,^(8)^ detected chromosomal abnormalities using quantitative fluorescence polymerase chain reaction in 53 of 175 cases (30.2%), with a cut-off value for NT of ≥3.5 mm (99^th^ percentile for 11-13^6^ gestational weeks). In their study, 20 cases (17.5%) had structural defects with normal karyotypes. Lithner et al.,^(9)^ observed abnormal karyotypes in 164 of 341 cases with increased NT (48%), whereas 12 of 139 cases with normal karyotypes had structural anomalies (8.6%). In our study, the cut-off value for NT was ≥1.5 MoM; abnormal karyotypes were present in 21 of 78 cases (26.9%) and structural anomalies were diagnosed in 12 of 57 cases with normal karyotypes (21.05%).

Cardiac defects are the most common fetal structural defects, both in the general pregnant population and in the population with increased NT. The prevalence of major cardiac defects increases with increasing NT values^(10)^. An NT thickness ≥3.5 mm in a chromosomally normal fetus has been correlated with a prevalence of congenital cardiac anomalies of 23 per 1000 pregnancies.^(11)^ In another study, the prevalence of congenital heart disease was about 0.5% in fetuses with NT < median; 1% for NT between median and 95^th^ percentile, 2% for NT between 95^th^ and 99^th^ percentiles, and increased to 3.5%, 6.5%, and 12.5% for NT of 3.5-4.4 mm, 4.5-5.4 mm, and ≥5.5 mm, respectively^(12)^.

In our study, an increased NT was accepted as ≥1.5 MoM, and cardiac anomalies were observed in 9.6% (5/57) of fetuses with normal karyotypes, similar to the rate reported by Muller et al.,^(13)^ higher than that found by Mavrides et al.,^(14)^ and lower than that reported by Hyett et al^(15)^. If we had taken the cut-off value as 3.5 mm, cardiac anomaly rate in fetuses with increased NT and normal karyotypes would be 11.8% (4/34); however, one case would be missed with an NT measurement of 2.4 mm.

In the current study, intrauterine exitus occurred in two fetuses (2.5%) with neither abnormal karyotypes nor structural defects. Lithner et al.,^ (9)^ also reported one case of intrauterine exitus (0.7%) with a normal karyotype and detailed scanning results.

The overall incidence of fetal anomalies or loss in our study was 44.8% (35/78). We defined ≥1.5 MoM as increased NT, and observed a higher risk for anomalies or loss in fetuses above this cut-off compared with other studies that reported adverse outcome rates ranging between 11% and 25.9% for fetuses with increased NT, even though their cut-offs were most probably higher than ours in millimeters because of the differences in the definitions of increased NT.^(9,16,17,18)^ Abnormal fetal karyotype, congenital structural anomalies, and intrauterine fetal death were accepted as adverse fetal outcomes in our study. Souka et al.,^(16)^ observed chromosomal anomalies in 64.45% of fetuses with NT measurements ≥6.5 mm, and when karyotype was normal, the rate of live births with no defects in this group was 31.2%. According to the ROC curve analysis in our study, cases with NT >7 mm or NT MoM >4.27 invariably resulted in adverse fetal outcomes.

The preterm birth rate in the current study was 13.9%, including four multiple pregnancies. The preterm birth rate for singletons at our center is 12%, compared with 12.6% among pregnancies subjected to invasive procedures, including multiple pregnancies.^(19,20)^ These data suggest that increased NT did not significantly increase the preterm birth rate. Pihl et al.,^(21)^ observed a significant relationship between increased NT and preterm birth, with a cut-off value ≥2*.*0 mm, and Krantz et al.,^ (22)^ found a significant relation between NT ≥1*.*96 MoM (99^th^ centile) and preterm birth (*<*34 weeks). However, Dugoff et al.^(23)^ found no relationship between increased NT and preterm birth (*<*37 or ≤32 weeks).

Some studies in the literature have investigated the association between NT and birth weight^(22,24,25,26,27,28)^. Earlier studies found no relationship between NT and SGA^(22)^. However, more recent studies have revealed an association between NT and SGA/LGA. Poon et al.,^(24)^ demonstrated that birth weight increased with increasing fetal NT, and Papastefanou et al.^(26)^ found that the difference between the observed and expected values of NT was related to both SGA and LGA. Kelekci et al.,^(27)^ reported increased NT in macrosomic fetuses, whereas Weissmann-Brenner et al.,^(28)^ found a correlation between increased NT and LGA neonates in term non-diabetic patients. In our study, the rates of SGA and LGA among cases without fetal anomalies or loss, despite increased NT, were 13.9% and 4.7%, respectively. The apparent discrepancies between these rates and those of other recent studies may be attributable to the small sample sizes.

We were unable to diagnose any specific syndromes in our study group, but we speculate that they could have existed among the fetuses terminated for multiple anomalies. Furthermore, we only performed conventional karyotyping and did not conduct array comparative genomic hybridization (aCGH) or other advanced genetic tests. However, a previous systematic review and meta-analysis of 17 publications found that genomic microarray analysis identified a 5% incremental yield in fetuses with increased NT and normal karyotype^(29)^. This technology could thus help to explain the genetic basis of some of the congenital anomalies and intrauterine deaths.

Increased first-trimester NT necessitates conventional fetal karyotyping, followed by aCGH in cases with normal karyotype results if possible. In recent years, cell-free DNA in maternal serum has appeared to be a highly sensitive screening modality for aneuploidies; however, it is not sufficient in the evaluation of increased first-trimester NT due to the need for confirmation of the positive results and the possibility of false negatives, and also its wide spectrum of etiologies. Whole-exome sequencing may become the preferred technique for investigating fetuses with normal karyotypes and/or normal aCGH in the future. Early detailed scanning and fetal echocardiography between the 14^th^ and 16^th^ gestational weeks are advised in the event of increased NT, and both scans should be repeated at gestational week 18-22 if the results are normal. According to the ROC curves in our study, the optimal cut-off values for NT measurements for predicting fetal anomalies or loss were 3.05 mm and 2.02 MoM. Given that one out of five fetuses with cardiac anomalies in our series had an NT thickness <3.5 mm, we suggest that fetal echocardiography could be considered for NT thickness ≥3.5 mm, which corresponds to the 99^th^ percentile at 11-13^6^ gestational weeks, and for all cases with NT thickness ≥1.5 MoM. Infection screening might also be an option for fetuses with increased first trimester NT measurements, though infections are not frequently diagnosed, and a rate of only 1.4% was reported by Sebire et al.^(30)^ Although, few studies in the literature indicate that apparently normal newborns with increased first trimester NT and normal karyotype do not differ from the general population regarding long-term adverse outcomes,^(31)^ more long-term studies may provide further valuable information.

## Conclusion

NT measurement is considered to be an important tool in antenatal follow-up. Higher NT measurements are associated with poorer pregnancy outcomes according to our study as well as the studies in the literature. We have observed that all cases with NT measurements >7 mm or NT MoM >4.27 had either chromosomal or structural anomalies or resulted with intrauterine fetal exitus. Our study also indicates that fetal echocardiography must be considered as a part of the assessment for all cases of NT thickness ≥1.5 MoM.

## Figures and Tables

**Table 1:**
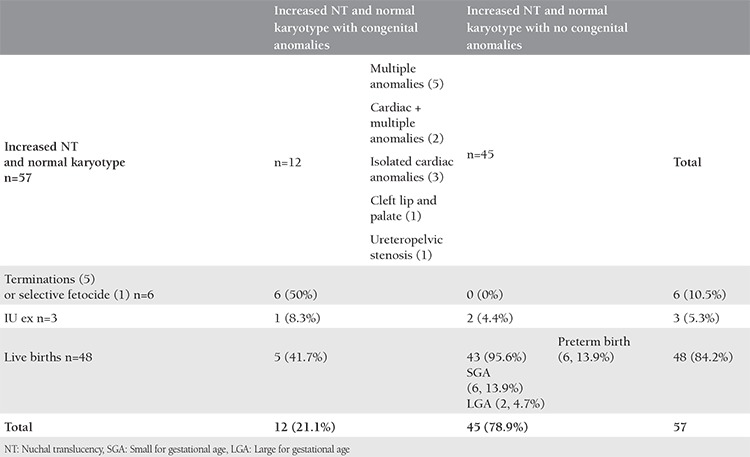
Pregnancy outcomes in cases with increased NT and normal karyotype

**Table 2:**
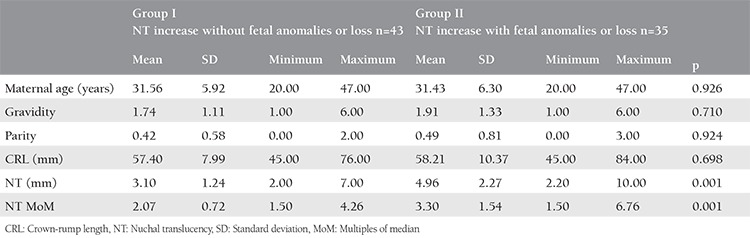
Demographic features, crown-rump length, and nuchal translucency in two increased NT groups without and with fetal anomalies or loss

## References

[1] Nicolaides KH, Sebire NJ, Snijders RJ (1999). Nuchal translucency and chromosomal defects. In: Nicolaides KH, Sebire NJ, Snijders RJ, editors. The 11-14-week scan: The diagnosis of fetal abnormalities. 1999:6-10 Parthenon.

[2] Nicolaides KH (2004). Nuchal translucency and other first trimester sonographic markers of chromosomal abnormalities. Am J Obstet Gynecol.

[3] Nicolaides KH, Sebire NJ, Snijders RJ (1999). Pathophysiology of increased nuchal translucency. In: Nicolaides KH, Sebire NJ, Snijders RJ, editors. The 11-14-week scan: The diagnosis of fetal abnormalities.

[4] Desilets V, Audibert F (2013). Investigation and management of nonimmune fetal hydrops. J Obstet Gynaecol Can.

[5] Kagan KO, Avgidou K, Molina FS, Gajewska K, Nicolaides KH (2006). Relation between increased fetal nuchal translucency thickness and chromosomal defects. Obstet Gynecol.

[6] Souka AP, Von Kaisenberg CS, Hyett JA, Sonek JD, Nicolaides KH (2005). Increased nuchal translucency with normal karyotype. Am J Obstet Gynecol.

[7] Pandya PP, Brizot ML, Kuhn P, Snijders RJ, Nicolaides KH (1994). Firsttrimester fetal nuchal translucency thickness and risk for trisomies. Obstet Gynecol.

[8] Pan M, Han J, Zhen L, Yang X, Li R, Liao C, et al (2016). Prenatal diagnosis of fetuses with increased nuchal translucency using an approach based on quantitative fluorescent polymerase chain reaction and genomic microarray. Eur J Obstet Gynecol Reprod Biol.

[9] Lithner CU, Kublickas M, Ek S (2016). Pregnancy outcome for fetuses with increased nuchal translucency but normal karyotype. J Med Screen.

[10] Hyett J (2004). Does nuchal translucency have a role in fetal cardiac screening?. Prenat Diagn.

[11] Nicolaides KH (2004). Nuchal translucency and other first trimester sonographic markers of chromosomal abnormalities. Am J Obstet Gynecol.

[12] Atzei A, Gajewska K, Huggon IC, Allan L, Nicolaides KH (2005). Relationship between nuchal translucency thickness and prevalence of major cardiac defects in fetuses with normal karyotype. Ultrasound Obstet Gynecol.

[13] Muller MA, Clur SA, Timmerman E, Bilardo CM (2007). Nuchal translucency measurement and congenital heart defects: modest association in low-risk pregnancies. Prenat Diagn.

[14] Mavrides E, Cobian-Sanchez F, Tekay A, Moscoso G, Campbell S, Thilaganathan B, et al (2001). Limitations of using first-trimester nuchal translucency measurement in routine screening for major congenital heart defects. Ultrasound Obstet Gynecol.

[15] Hyett J, Perdu M, Sharland G, Snijders R, Nicolaides KH (1999). Using fetal nuchal translucency to screen for major congenital cardiac defects at 10-14 weeks of gestation: population based cohort study. BMJ.

[16] Souka AP, Krampl E, Bakalis S, Heath V, Nicolaides KH (2001). Outcome of pregnancy in chromosomally normal fetuses with increased nuchal translucency in the first trimester. Ultrasound Obstet Gynecol.

[17] Ayras O, Tikkanen M, Eronen M, Paavonen J, Stefanovic V (2013). Increased nuchal translucency and pregnancy outcome: a retrospective study of 1063 consecutive singleton pregnancies in a single referral institution. Prenat Diagn.

[18] Bilardo CM, Muller MA, Pajkrt E, Clur SA, van Zalen MM, Bijlsma EK (2007). Increased nuchal translucency thickness and normal karyotype: time for parental reassurance. Ultrasound Obstet Gynecol.

[19] Yanık Ff, Gülümser Ç, Şahin Uysal N. The problem of preterm delivery in twin pregnancies. 13th World Congress in Fetal Medicine, Abstracts, Preterm birth, 2014 Poster.

[20] Şahin Uysal N, Gülümser Ç, Yanık Ff. Gebelikte İnvaziv Girişim Yapılan Hastalarda Preterm Doğum Oranı. Türkiye Maternal Fetal Tıp ve Perinatoloji Derneği IX. Ulusal Kongresi 2014, Poster.

[21] Pihl K, Sørensen TL, Nørgaard-Pedersen B, Larsen SO, Nguyen TH, Krebs L, et al (2008). First-trimester combined screening for Down syndrome: prediction of low birth weight, small for gestational age and pre-term delivery in a cohort of non-selected women. Prenat Diagn.

[22] Krantz D, Goetzl L, Simpson JL, Thom E, Zachary J, Hallahan TW, et al (2004). Association of extreme first-trimester free human chorionic gonadotropin-beta, pregnancyassociated plasma protein A, and nuchal translucency with intrauterine growth restriction and other adverse pregnancy outcomes. Am J Obstet Gynecol.

[23] Dugoff L, Hobbins JC, Malone FD, Porter TF, Luthy D, Comstock CH, et al (2004). First-trimester maternal serum PAPP-A and free-beta subunit human chorionic gonadotrophin concentrations and nuchal translucency are associated with obstetric complications: a population-based screening study (the FASTER Trial). Am J Obstet Gynecol.

[24] Poon LC, Karagiannis G, Staboulidou I, Shafiei A, Nicolaides KH (2011). Reference range of birth weight with gestation and firsttrimester prediction of small-for-gestation neonates. Prenat Diagn.

[25] Poon LC, Karagiannis G, Stratieva V, Syngelaki A, Nicolaides KH (2011). First-trimester prediction of macrosomia. Fetal Diagn Ther.

[26] Papastefanou I, Souka AP, Pilalis A, Eleftheriades M, Michalitsi V, Kassanos D (2012). First trimester prediction of small- and large-forgestation neonates by an integrated model incorporating ultrasound parameters, biochemical indices and maternal characteristics. Acta Obstet Gynecol Scand.

[27] Kelekci S, Yilmaz B, Savan K, Sonmez S (2005). Can increased nuchal translucency in the first trimester of pregnancy predict gestational diabetes mellitus. J Obstet Gynaecol.

[28] Weissmann-Brenner A, Weisz B, Lerner-Geva L, Gindes L, Achiron R (2011). Increased nuchal translucency is associated with large for gestational age neonates in singleton pregnancies. J Perinat Med.

[29] Grande M, Jansen FA, Blumenfeld YJ, Fisher A, Odibo AO, Haak MC, et al (2015). Genomic microarray in fetuses with increased nuchal translucency and normal karyotype - a systematic review and metaanalysis. Ultrasound Obstet Gynecol.

[30] Sebire NJ, Bianco D, Snidjers RJM, Zuckerman M, Nicolaides KH (1997). Increased fetal nuchal translucency thickness at 10-14 weeks: is screening for maternal-fetal infection necessary?. Br J Obstet Gynaecol.

[31] Iuculano A, Pagani G, Stagnati V, Floris M, Ibba RM, Monni G (2016). Pregnancy outcome and long-term follow-up of fetuses with isolated increased NT: a retrospective cohort study. J Perinat Med.

